# South Africa—Serious about Biodiversity Science

**DOI:** 10.1371/journal.pbio.0030145

**Published:** 2005-05-17

**Authors:** Michael Cherry

## Abstract

A new government act, the creation of several centres of excellence and an injection of funding all indicate that biodiversity science is thriving in South Africa.

In 1772, Carolus Linnaeus wrote a letter, now oft-quoted, to Ryk Tulbagh, the Governor of the Cape—in which he envied Tulbagh's “sovereign control of that Paradise on Earth, the Cape of Good Hope, which the beneficent Creator has enriched with his choicest wonders”. Two and a half centuries later, South Africa's biodiversity remains a great source of interest to the scientific community—and for good reason (Box 1). Plant biodiversity, with over 20 000 different species, is in the foreground: South Africa, which comprises less than 1% of the world's land surface, contains 8% of its plant species. Perhaps less well known is that the country also contains 7% of all bird, mammal, and reptile species, and 15% of known coastal marine species.

**Box 1. Biodiversity and the South African Economy**

The extraordinary diversity of habitats found on the southern tip of the African continent includes three globally recognized biodiversity hotspots: the temperate Cape Floristic Region (see Figure 1), the arid Succulent Karoo, and the subtropical Maputaland-Pondoland-Albany area. On account of its early colonization and relative wealth, South Africa has good universities, museums, and herbaria, and reasonably well-run conservation agencies at both the provincial and national levels. But in a country whose history has been characterized by fighting over land, the 6.6% of its land surface with formal conservation status (in other words, protected by the state) lags behind the global mean of 11.5%. By contrast, 17% of its coastline is formally protected.

Protection is important for a number of reasons. A decade after the advent of democracy, the economy is booming at last, with the country currently experiencing the longest sustained period of growth in its history since the early 1960s. Rising levels of affluence have led to increased demand for housing, roads, and recreational facilities—all developments that affect biodiversity. The benefits that biodiversity brings to the economy are increasingly being realized, notably through ecotourism. Tourism is the fastest growing sector of the economy, having risen to 7% of GDP in 2003, from only 2% a decade previously. The virtual abandonment of agriculture subsidies has led to much marginal agricultural land—previously farmed essentially to generate subsidy—being converted to private nature reserves, used either for ecotourism or for hunting, and sometimes for both. Such land now comprises 13% of the country's surface—more than twice the area protected by the state.

There are also direct benefits associated with harvesting indigenous flora and fauna. Some are quantifiable, such as the fishing industry, worth just over half a billion US dollars last year. Others cannot be measured directly, but are no less important for that. For example, almost 20% of South Africa's plants, or 3 689 species, are used as traditional medicines, which still provide the first resource for primary health care to almost three-quarters of South Africa's population. The challenge of sustainable harvesting is difficult enough when yields are known, but even more daunting when they are undetermined.

## The South African National Biodiversity Institute

South Africa's new Biodiversity Act, signed on September 1, 2004, expands the mandate of the National Botanical Institute (NBI) to include responsibilities relating to the full diversity of the country's fauna and flora; it is now known as the South African National Biodiversity Institute (SANBI) (Pretoria, South Africa). Previously responsible for eight national botanical gardens and three herbaria, as well as botanical research centres in Pretoria and at its largest garden at Kirstenbosch on the slopes of Table Mountain, it now additionally should influence the prospects of all collections of specimens; coordinate research on indigenous biodiversity and its sustainable use; advise conservation agencies and municipalities with regard to planning decisions relating to biodiversity; coordinate the control of invasive species; and monitor the effect of any genetically modified organisms released into the environment.

*“There are very few developing countries which have the prospect of delivering jobs related to the conservation industry. South Africa has this prospect.”*

**Figure 2:**
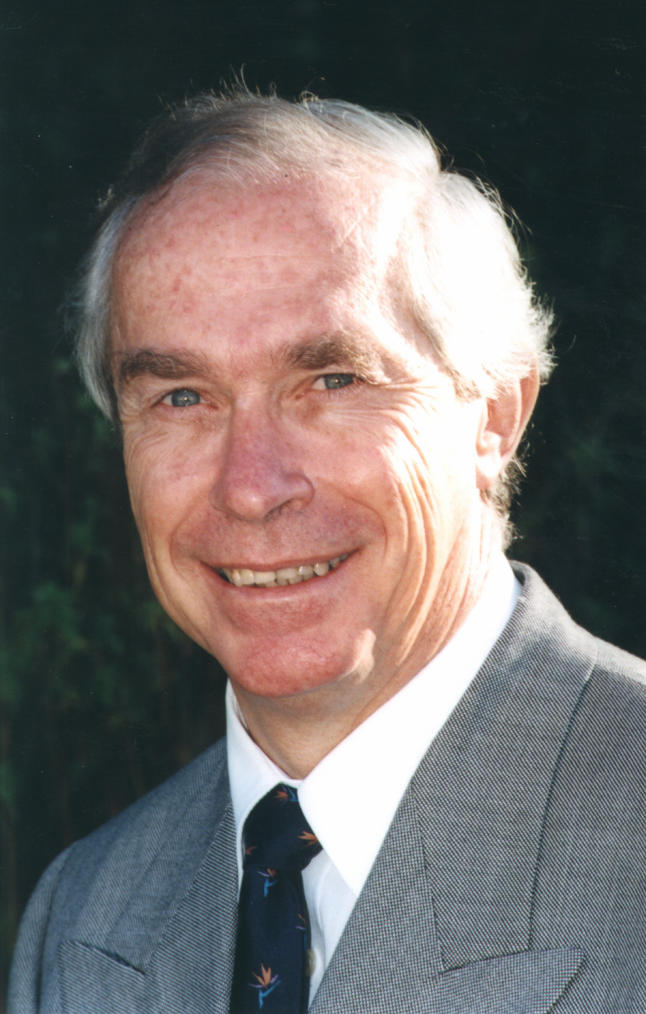
Brian Huntley, Acting Chief Executive of the South African National Biodiversity Institute (SANBI)

Acting Chief Executive Officer Brian Huntley (Figure 2) admits openly that this is quite a brief. It's not difficult to see why it is the former NBI that has inherited this mantle, since it has become, over the past decade, by far the largest and most dynamic South African institution working in the biodiversity arena. Operating under the aegis of the Department of Environment Affairs, it was formed in 1989 through the amalgamation of what had previously been the National Botanical Gardens and the Botanical Research Institute. Currently supporting 680 staff, it has flourished particularly during Huntley's tenure, which has been characterized by an influx of externally funded projects, to the extent that external income —$18 million per annum—now exceeds the $16 million it receives from its parliamentary grant and from entrance fees paid by the million or so visitors to its gardens each year. Huntley is optimistic that this brief can succeed, although he concedes that in few countries does any single institution bear responsibility for research, information dissemination, and applications relating to biodiversity. But he believes that South Africa is a small enough country, with enough good intellectual capacity, for this model to work.

This view is echoed by Andrew Balmford of Cambridge University, who is spending a sabbatical at the Percy Fitzpatrick Institute for African Ornithology at the University of Cape Town. “While the obvious challenge is to link biodiversity conservation to development needs”, he says, “there are very few developing countries which have the prospect of delivering jobs related to the conservation industry. South Africa has this prospect, not only because it is unbelievably diverse, but because of international goodwill towards the country”.

Huntley's strategy will be to bring a sound scientific base to the enterprise, as he has already done with the NBI. There are several examples of this. One is the African Plants Initiative—being led by the SANBI, Kew Gardens in the United Kingdom (London), and the United States Missouri Botanical Garden (St. Louis, Missouri, United States)—whose aim is to create an electronic library of the type specimens of all African plants: an estimated 300 000 accessions of 60 000 species. This includes scanned pictures of each individual specimen, the quality of which, according to Huntley, “is as good as if one were examining the specimen through a standard dissecting microscope.” Another example involves placing the 2.5 million specimens in South Africa's herbaria on a computerized database, a task now 40% complete. A third example is the Southern African Botanical Diversity Network (Pretoria, South Africa), founded in 1996, which has, to date, trained 200 botanists in ten countries in the region.

By contrast, research on zoological diversity, traditionally the domain of the country's natural history museums, has lagged behind. The Iziko South African Museum in Cape Town, for example, one of the country's five national natural history museums, now has only seven research staff in natural history compared to the 16 it had in 1989. Why have they failed to capitalize on external funding in the way the NBI has done? One answer is that, unlike the three national herbaria, which all fell under the jurisdiction of the NBI, these five institutions have retained their institutional autonomy, and consequently have remained fragmented in their efforts. One, the South African Institute for Aquatic Biodiversity (Grahamstown, South Africa), is run by the National Research Foundation, while the other four are funded by grants from the Department of Arts and Culture, which has tended to view them as educational, rather than research, organizations. Huntley emphasizes that the SANBI does not aspire “to do what other organizations are already doing well.” With regard to natural history museums, he says that the first step will be to take the initiative in conducting a thorough review of their funding, and the “best practice of dealing with large and dispersed collections”.

## Centres of Excellence in Biodiversity

Another related development is the recent announcement of the Department of Science and Technology that it will fund six centres of excellence nationally at South African universities, with effect from 2005. No fewer than three of these centres are focused on biodiversity: one at the Fitzpatrick Institute (Cape Town, South Africa), concerned with birds as models for understanding biodiversity processes; one at the University of Pretoria (Pretoria, South Africa), which will be concerned with pathogens on indigenous trees; and a third in the Faculty of Science at the University of Stellenbosch, which will focus on invasion biology. All of the centres are based at the host institution, but can disburse funds to collaborators elsewhere in the country.

*“These centres of excellence...are a manifestation of the seriousness with which the South African government is taking science”*

**Figure 3:**
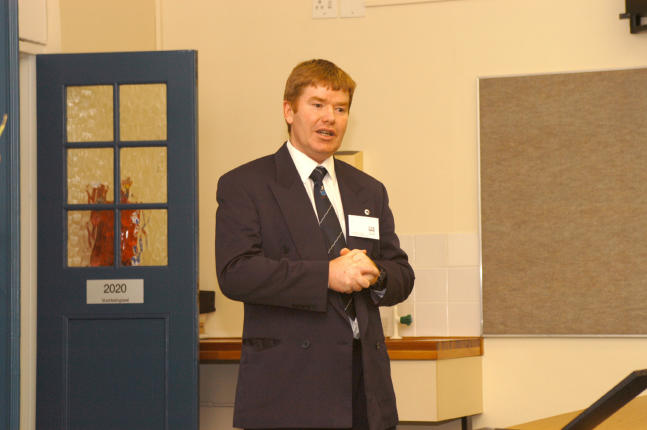
Steven Chown, Director of the Centre for Invasion Biology

These centres of excellence, says Steven Chown (Figure 3), director of the Centre for Invasion Biology, “are a manifestation of the seriousness with which the South African government is taking science”. Others are more sceptical. “I don't think that in the biodiversity field research is optimally conducted by large groups, but by smaller groups of collaborators”, says David Ward of the School of Biological and Conservation Science at the University of KwaZulu-Natal (KwaZulu-Natal, South Africa). “Unlike fields like nuclear physics, in ecology costs are relatively low—large centres just incur additional administrative costs, without improving the quality of the science produced”, he adds. Rob Slotow, from the same school, feels that the centres have confined their collaborative efforts to junior colleagues outside their own institutions. “There is very little real inter-institutional collaboration taking place at a senior level, which is disappointing”, he says, as “the opportunity to kick-start a different level of funding for biodiversity research in the country—the aim of the centres-of-excellence concept—has been missed”.

The research programme of the Fitzpatrick Institute is based on two interlinking themes: understanding and maintaining avian biodiversity. Tim Crowe will lead a group investigating the processes responsible for the origins of African biodiversity, which will investigate how the process of speciation in birds occupying disjunct distributions in habitats ranging from montane forest to desert may have been influenced by past biogeographic corridors that shifted with changing climates many millions of years ago.

**Figure 4:**
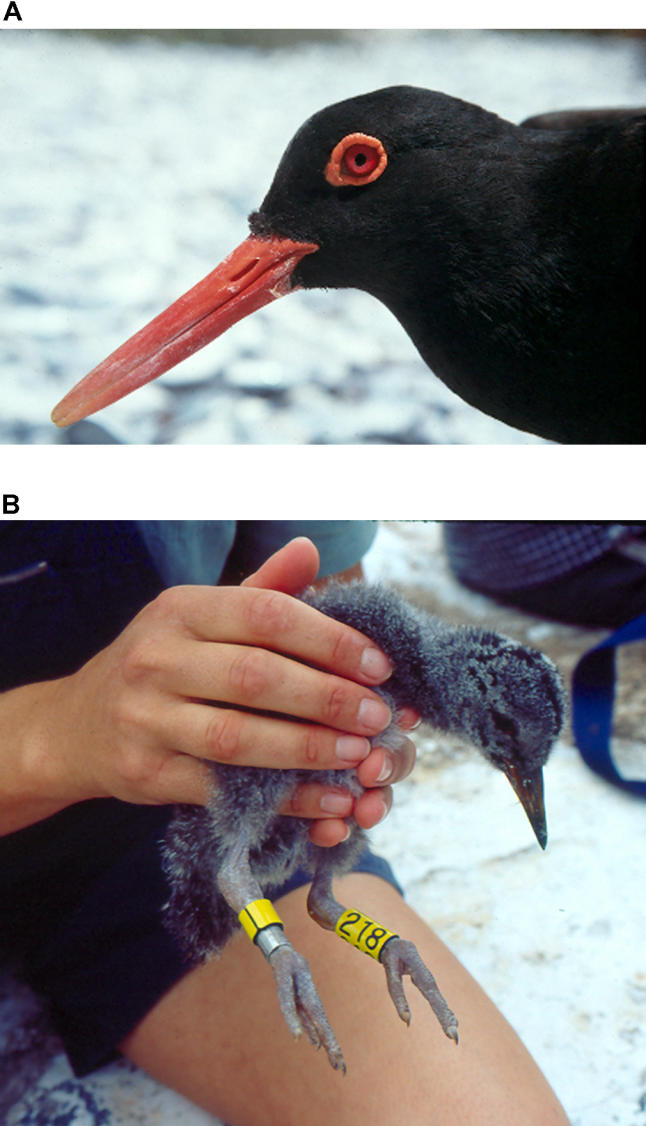
African Black Oystercatchers (A) Portrait of an adult African Black Oystercatcher. (Photo: Philip Hockey) (B) An African Black Oystercatcher chick with the numbered colour rings that are used to follow its survival and migratory movements over several years. (Photo: Doug Loewenthal)

Understanding how relationships between organisms and their environments influence the form and functioning of biological systems is the core focus of a second grouping of researchers. For example, Phil Hockey is studying life-history traits and movement patterns of African Black Oystercatchers (Figure 4A and 4B), where research to date indicates that migration in juveniles is facultative, responding to body condition. Oystercatcher populations are increasing, primarily as a consequence of a ban imposed several years ago on four-wheel-drive vehicles on beaches. These increasing populations provide a unique opportunity to test the hypothesis that migration in stable habitats evolves—initially in juveniles—in response to population density exceeding carrying capacity.

The Fitzpatrick Institute's teaching efforts have been impressive; its master's course in conservation biology has produced close to 150 graduates from 15 different African countries since its inception in 1992. But are birds really good models for understanding changes in patterns of biodiversity? Many would argue that they are not, since their mobility allows them to respond to environmental changes by colonizing new areas with relative ease. The centre's director, Morné du Plessis, counters that “while birds are often not good indicators of environmental change, they are a group for which good baseline information exists, as well as being relatively easy to study.”

The centre for Tree Health Biotechnology forms part of the Forestry and Agriculture Biotechnology Institute at the University of Pretoria. The institute has to date focused largely on pathogens on trees used in commercial forestry, most of which are alien species, but according to its director, Mike Wingfield, the centre will be devoted specifically to studying pathogens on indigenous trees. But the two, he adds, are closely related. “Alien trees used in commercial forestry are often able to thrive because they are distanced from their natural pathogens”, he says, but “we are now observing natural pathogens of native trees switching hosts to alien species”. This happens usually when alien and native trees are reasonably closely related. Wingfield believes that an example is the fungus causing *Cryphonectria* canker, which he and his collaborators have recently shown, on the basis of DNA sequences, to occur on both the native waterberry tree, *Syzgium cordatum*, and on the alien *Eucalyptus* (widely used for forestry in South Africa), from which it was first reported in 1989.

**Figure 5:**
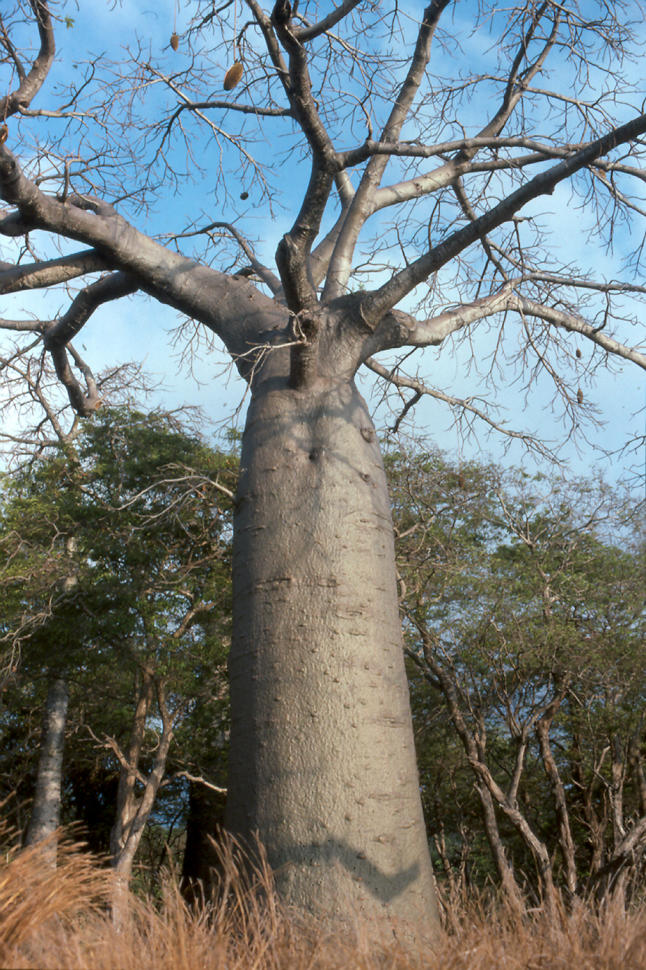
African Baobab The African baobab, *Adansonia digitata*, can reach up to 10 meters in diameter and can live more than 1 000 years. (Photo: Peter Jones)

Similarly, native trees, which are often of importance to local communities, could be at risk from pathogens imported on alien species. The kiaat tree *Pterocarpus angolensis*, for example, is widely used by wood-carvers, as well as in traditional medicines. Trees are reported to be dying, but it is unknown whether this is on account of pathogens, climate change, or changing fire regimens. There have also been sporadic reports of dying baobabs (Figure 5)—one of the icons of the African savannah—over the past 15 years. Wingfield says that “while at the present time, there is no clear evidence that an unknown fungus is killing baobabs, these reports should not go unheeded”. “Both kiaat and baobab deaths merit attention, which the centre should now be able to provide,” says Wingfield.

The Centre for Invasion Biology is somewhat different from the other two centres in that it focuses on a specific question, namely how invasions affect biodiversity and ecosystem functioning. Of its annual budget of $1 million, five-sixths will come from the government, with the remainder being provided by the University of Stellenbosch. Chown believes that this is a bargain, considering the magnitude of the problem: the global cost of addressing biological invasions is estimated to be $1.4 trillion annually—about 5% of global GDP.

In South Africa, invasive plants are a particular problem. Apart from the threat they pose to indigenous diversity, they are a fire hazard in several ecosystems in which burning is part of the natural cycle—and perhaps most importantly, they are a huge drain on water in a country in which this is a scarce commodity. This has led to a programme—certainly the largest of its kind in any developing country—called Working for Water, in which unemployed persons have been hired to conduct alien clearances on a large scale. Chown's centre will provide policy inputs to the programme as part of a broad range of pure and applied research objectives.

The Centre for Invasion Biology will address both long-term studies of invasive organisms in different habitats and the outcomes of remediation programmes, which Chown views as large-scale ecological experiments whose effects need to be studied. “The Working for Water rehabilitation programme provides excellent opportunities for understanding relationships between changes in species richness and changes in ecosystem function, and how alien invasion and clearance affects both phenomena”, he says.

A second component will attempt to study invasions from the outset, as opposed to post hoc. Chown proposes to investigate concomitant climate and land-use changes in the Cedarberg mountains, a range 200 km north of Cape Town. The predominant land-use patterns of agriculture and ecotourism are changing rapidly in the area, which he predicts will be accompanied by changes in the extent and identity of invasive species. Additionally, climate-change models predict that this relatively arid part of the fynbos biome (the major vegetation type of the Cape Floristic Region) will be transformed within 50 years into a semi-desert system.

To what extent will these different ventures find a common purpose? There are some obvious links: Huntley sits on the board of the Fitzpatrick Institute, whose master's course in conservation biology has supplied many graduates to the NBI over the past 15 years. Chown sits on the board of the SANBI, together with representatives of the departments of Science and Technology, Agriculture and Environment Affairs, and David Mabunda, chief executive officer of South African National Parks. As the chief executive of the SANBI now exercises a huge degree of statutory influence over the nation's biodiversity, the answer to this question is closely related to that of who will replace Huntley, who is now 61. Huntley's tenure will be a hard act to follow, and the future of South Africa's biodiversity will lie largely in the hands of his successor.

**Figure 1:**
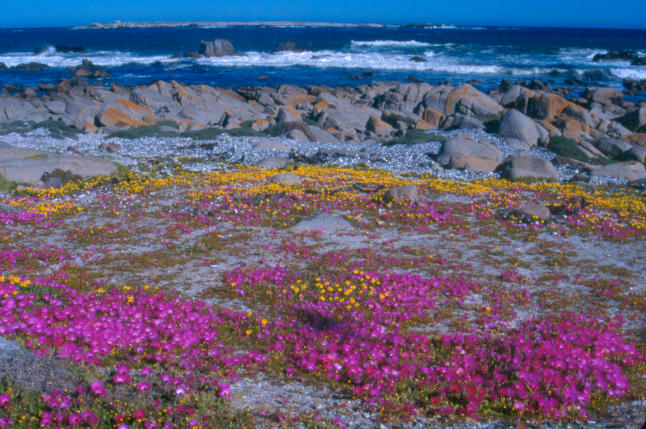
Cape Flowers in August (Photo: Peter Jones)

**Where to Find Out More**

South African National Biodiversity Institute (SANBI): http://www.nbi.ac.za/

Percy Fitzpatrick Institute of African Ornithology: http://www.fitzpatrick.uct.ac.za/

DST Centre of Excellence for Invasion Biology: http://academic.sun.ac.za/cib/

Centre of Excellence in Tree Health Biotechnology: http://fabinet.up.ac.za/CoE/

Working for Water Programme: http://www-dwaf.pwv.gov.za/wfw/

## Abbreviations

NBI: National Botanical Institute
SANBI: South African National Biodiversity Institute

